# Presumptive Diagnosis of Pallister-Hall Syndrome Using Magnetic Resonance Imaging

**DOI:** 10.7759/cureus.21735

**Published:** 2022-01-30

**Authors:** Yusuf Mehkri, Krishna Surapaneni, Bedirhan Tarhan, Tiffany Eisenbach, Ahmet Bilgili, Ibrahim S Tuna, Hans H Shuhaiber, Kwame Anyane-Yeboa

**Affiliations:** 1 Neurosurgery, University of Florida College of Medicine, Gainesville, USA; 2 Neuroradiology, Austin Radiological Association, Austin, USA; 3 Neurology, University of Florida College of Medicine, Gainesville, USA; 4 Radiology, University of Florida College of Medicine, Gainesville, USA; 5 Pediatrics, Columbia University Irving Medical Center, New York, USA

**Keywords:** genetic disorder, bifid epiglottis, hypothalamic hamartoma, magnetic resonance imaging, mri, pallister-hall syndrome

## Abstract

Pallister-Hall syndrome (PHS) is an extremely rare genetic disorder for which the diagnosis is often overlooked. The objective of this case report is to highlight how clinical features used in conjunction with brain MRI findings can lead to an expeditious diagnosis without the need for invasive measures or genetic test results.

We present the case of a three-day-old infant delivered at 34 and 4/7 weeks gestation who presented with mild respiratory distress and bilious emesis in the setting of an uncomplicated gestational course and vaginal delivery with no known teratogen exposure. A diagnosis of Pallister-Hall syndrome was made on the basis of physical exam findings, hormonal abnormalities and the identification of a hypothalamic hamartoma on brain MRI. The patient underwent multiple procedures for diagnosis and management of PHS complications, including a diverting jejunostomy for a long-segment Hirschsprung’s and a laryngoscopy which identified a bifid epiglottis. The patient tolerated the interventions and did not have seizures on admission.

The MRI brain detection of a hypothalamic hamartoma led to an earlier diagnosis of Pallister-Hall syndrome and thus further screening and identification of complications associated with this disorder were performed before genetic analyses or brain biopsies were obtained. Given the unique MRI features of hypothalamic hamartomas, brain MRI can be a useful tool for making an early PHS diagnosis when taken with clinical features concerning possible PHS.

## Introduction

Pallister-Hall syndrome (PHS) is a pleiotropic disorder first described in 1980 and characterized by hypothalamic hamartoma, central or postaxial polydactyly, hypopituitarism, and variable visceral anomalies [1]. It is inherited in an autosomal dominant manner with both variable inter- and intra-familial expressivity [1]. More recently in 1990, Gorlin et al. re-examined the syndrome and added bifid epiglottis, dysplastic nails, and renal anomalies as additional major signs [2]. In 2005, mutations in the zinc-finger transcription factor gene (GLI3) on the chromosomal locus 7p13 were found to be the causative factor in 95% of patients with clinically suspected PHS [3]. The GLI3 protein functions in the sonic hedgehog-signalling pathway, and plays a complex role and is widely expressed in embryogenesis, especially in the development of the central nervous system (CNS) and many other organ systems. The purpose of this report is to illustrate that MRI can prospectively suggest the diagnosis of PHS in the appropriate clinical setting before more sophisticated genetic and molecular analysis and avoid unnecessary neurosurgical biopsy at the time of initial presentation.

## Case presentation

**Figure 1:**
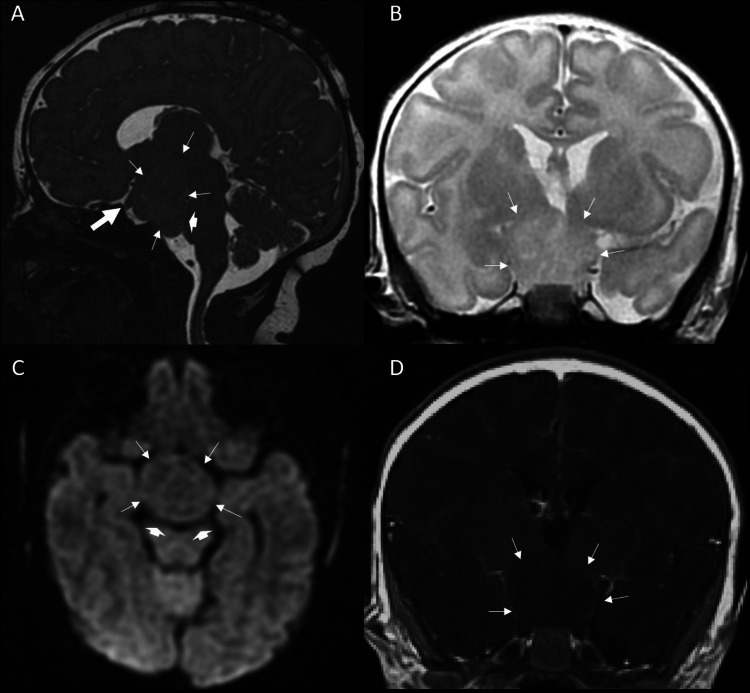
First week of life brain MRI A: Sagittal 3DT2, B: Coronal T2W, C: Axial DWI, and D: Coronal postcontrast T1W of the brain MRI in the first week of life demonstrates large suprasellar mass (outlined by the arrows) compatible with hamartoma. The signal characteristics within the hamartoma on T1W (D) and T2W images (A and B) are identical to the intensities of the normal grey matter and the non-myelinated white matter. The lesion involves the hypothalamus and fills the suprasellar, perimesensephalic and upper prepontine cisterns. There is a mild mass effect upon the optic chiasm which is anteriorly and superiorly displaced (thick arrow on A). There is also a mass effect upon the midbrain and pons, causing flattening of the cerebral peduncles (arrowheads on C) and ventral pons (arrowhead on A). Postcontrast imaging demonstrates no abnormal enhancement (D). DWI characteristics are also similar to the brain without evidence of hypercellular process (C). MRI = Magnetic resonance imaging, 3DT2 = 3 dimensional T2, T2W = T2 weighted, DWI = Diffusion weighted imaging, T1W = T1 weighted

**Figure 2:**
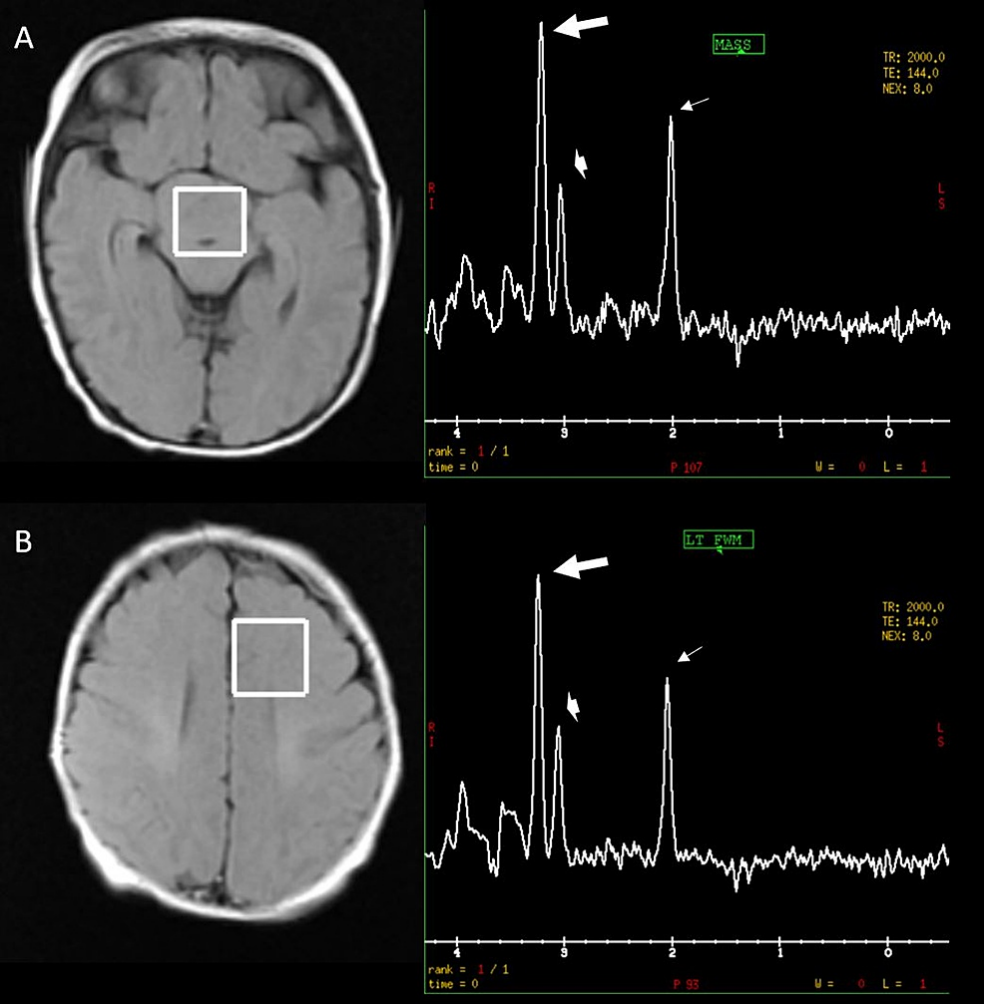
Single-voxel proton MR spectroscopy Single voxel MR Spectroscopy with TE=144 msec, obtained from the hypothalamic lesion (A) and left frontal lobe (B) demonstrates similar metabolites including choline (thick arrow at 3.2ppm), creatine (arrowhead at 3.0ppm) and NAA (arrow at 2.0ppm) appropriate for the patient’s age. No abnormal elevation of choline peak is seen within the lesion to suggest a more aggressive lesion. No abnormal lactate peak at 1.3ppm is seen to suggest necrosis or ischemia. MR = Magnetic resonance

The neonatal patient was the product of an uncomplicated pregnancy and normal spontaneous vaginal delivery at 34 and 4/7 weeks to an otherwise healthy 19-year-old G1P1 at an outside institution. Prenatal labs were normal and no maternal exposure to drugs, alcohol, or other teratogens was known. Apgar scores were 9 and 10 at one and five minutes, respectively, and the initial birthweight was 3105 grams. On newborn exam, the patient was found to have bilateral polydactyly, microphallus, and mild respiratory distress. On day of life three, the patient was found to have bilious emesis and an abdominal X-ray suggestive of small bowel obstruction. A follow-up contrast enema showed no visualization of the colon past the hepatic flexure, and contrast was not seen past the jejunum on an upper gastrointestinal study. Subsequently, the patient underwent exploratory laparotomy, rectal and intestinal biopsies, and diverting jejunostomy with a mucus fistula without bowel resection. Pathology results were consistent with a long-segment Hirschsprung’s disease. The patient was transferred to our institution for further workup and long-term management. Further evaluation and testing demonstrated the patient’s karyotype to be 46XY, with low growth hormone and testosterone. An MRI was completed to evaluate for etiologies of the patient's hypopituitarism. The MRI demonstrated a large non-enhancing prepontine mass measuring 26 x 19 mm causing superior displacement of the optic chiasm and mildly deforming the ventral pons (Figure 1). The signal characteristics of the mass closely followed that of gray matter on T2 and diffusion-weighted images. Single-voxel proton MR spectroscopy (TE=144 msec) was performed which demonstrated no significant discrepancy in spectroscopic characteristics of the mass compared to control-voxel imaging of the adjacent left frontal white matter (Figure 2).

**Figure 3:**
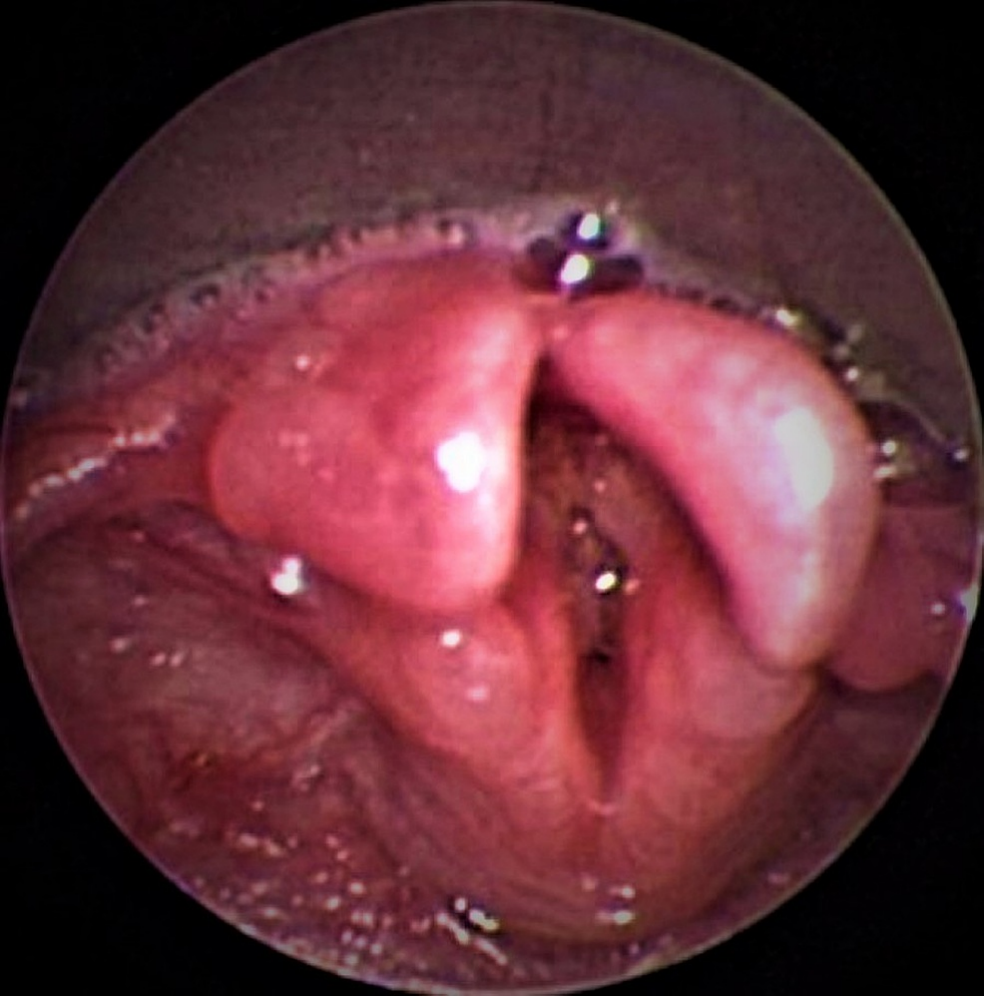
Laryngoscopy demonstrating bifid epiglottis

A renal ultrasound showed no evidence of congenital anomalies. Laryngoscopy was performed to further evaluate possible anatomic causes of the patient’s irregular breathing pattern, which had a stuttering pattern on inspiration and nasal congestion that transmitted to the chest. Laryngoscopy demonstrated a bifid epiglottis and tracheomalacia (Figure 3). The patient did not have any witnessed gelastic seizures during a protracted hospital course and continuous scalp electrode electroencephalogram recordings were normal.

## Discussion

Pallister-Hall syndrome is an extremely rare syndrome initially characterized in 1980 by Hall et al. as a neonatally lethal malformation syndrome with a wide range of abnormalities primarily associated with hypothalamic hamartoma (HH), mesoaxial polydactyly, postaxial polydactyly types A and B and bifid epiglottis [1,4]. Affected individuals may also display an imperforate anus, renal abnormalities, genitourinary anomalies, pulmonary segmentation anomalies, and additional skeletal anomalies [4].

In 1996, the National Institute of Health held a workshop to characterize PHS, establishing the diagnostic criteria for PHS which included both HH and mesoaxial polydactyl [5]. The criteria for diagnosing sub-PHS, which is a descriptor applied to individuals who have features of PHS but do not meet the complete diagnostic criteria for PHS, was also established. To have a sub-PHS diagnosis, patients must display either mesoaxial polydactyly, HH, oligodactyly, or postaxial polydactyly in conjunction with one of the following: bifid epiglottis, imperforate anus, small nails, hypopituitarism, growth hormone deficiency, genital hypoplasia. Furthermore, identification of a heterozygous pathogenic variant in GLI3 by molecular genetic testing lends support to both a PHS and sub-PHS diagnosis [4].

Pallister-Hall syndrome is inherited in an autosomal dominant pattern typically due to a GLI3 frameshift gene mutation. Approximately 95% of PHS patients show a mutation in the GLI3 gene in the short arm of chromosome 7, which has variable penetrance and expressivity resulting in a spectrum of abnormalities. Around 75% of individuals diagnosed with PHS have an affected parent, and it affects males and females equally [6,7]. Potentially, the variable penetrance is the reason for the sub-PHS clinical presentation rather than the heterozygous variant since it is an autosomal dominant disease.

The true prevalence of PHS is unknown and only a handful of patients have been identified in the medical literature. Due to these reasons, it is commonly underdiagnosed or misdiagnosed for similarly presenting conditions such as non-syndromic postaxial polydactyly Type-A [4]. An initial warning sign for a physician that a patient may present with PHS is the presence of polydactyly. However, polydactyly itself is not an uncommon phenomenon in newborn children [8]. Some conditions that can also present with polydactyly include McKusick-Kaufman syndrome (autosomal recessive, hydrometrocolpos, and congenital heart defects), oral-facial-digital syndrome type VI (autosomal recessive, cerebellar vermis hypoplasia, and tongue hamartomas), Ellis-van Creveld syndrome (autosomal recessive, congenital heart defects and skeletal dysplasia), Smith-Lemli-Opitz syndrome (autosomal recessive, hypocholesterolemia, and multiple cardiac and visceral anomalies), and Greig cephalopolysyndactyly syndrome (autosomal dominant and craniofacial abnormalities).

The presence of a HH, however, helps in differentiating PHS from other genetic polydactyly syndromes with overlapping features [5]. Hypothalamic hamartomas are a rare condition occurring in nearly 1 to 100,000 children and are not pathognomonic for PHS. They may occur either as an isolated sporadic lesion or in association with other focused abnormalities such as PHS [9,10]. Each group of syndromes have a distinct clinical phenotype. Patients with isolated HHs also tend to have severe seizures, as well as severe cognitive, behavioral, and endocrine disorders. They may also have different types of seizures that are frequent and difficult to control with multiple anti-epileptic drugs (AEDs), even with surgical interventions. However, those with HHs associated with PHS have a more benign neurologic course and less severe symptomology. They may not have seizures or do have seizures that usually respond well to AEDs [11,12]. Moreover, an increasing body of research has also found individuals with significantly less severe phenotypic manifestations, and even some patients with normal pituitary function and a normal lifespan in patients with PHS associated HH [13].

Whereas clinical symptoms clue into a diagnosis of HH, brain MRI is a key diagnostic tool for accurate identification and classification of a HH [14]. The characteristic MRI features of hypothalamic hamartomas help differentiate them from more common lesions that may include craniopharyngioma, optic gliomas, hypothalamic gliomas and gangliogliomas. In contrast to HH, all of these tumors generally show contrast enhancement. There is no enhancement on contrast due to the blood-brain barrier. However, on rare occasions, enhancement has been reported [15]. Craniopharyngiomas account for approximately half of all suprasellar neoplasms in children and are the most common nonglial brain tumor in pediatric patients. Craniopharyngiomas are often heterogenous lesions containing cystic and solid enhancing components with focal calcifications. Suprasellar gliomas are less common and typically have variable enhancement with T2 hyperintensity and hypointense to isointense T1 signal on MRI. Unlike other lesions, hypothalamic hamartomas often have well marginated homogenous soft tissue masses iso-intense to the grey matter. Imaging characteristics of the HH significantly reduce the differential in hypothalamic/suprasellar lesions. [16,17].

Treatments also vary from patient to patient between isolated HHs and HHs associated with PHS. Patients with PHS present with a variety of additional symptoms that must be accounted for when considering any treatment options. Hypothalamic hamartomas are rarely surgically resected in a PHS patient. However, those with isolated HH generally undergo resection for seizure cessation. Additional treatment options include antiepileptic drugs, gonadotropin-releasing hormone agonists, gamma knife radiosurgery, and stereotactic thermal ablation [10].

## Conclusions

Given the unique clinical presentation of PHS and the rarity of hypothalamic hamartomas themselves, clinical criteria taken together with characteristic hypothalamic hamartoma MRI findings can help make a PHS diagnosis without the need for invasive measures such as brain biopsy and allows for a diagnosis to be made more quickly in comparison to using genetic and molecular analysis measures. As per the 1996 NIH diagnostic criteria, both the PHS and even sub-PHS diagnoses can be made based on physical exam features, hormonal abnormalities, and the presence of a hypothalamic hamartoma alone. As hypothalamic hamartomas are easily distinguished radiologically from other common pediatric suprasellar lesions based on their lack of contrast enhancement, they are an easily identifiable component of making a PHS diagnosis. Allowing for an easier and quicker PHS diagnosis is of utility as these children can then be screened for other PHS complications that may have not already been detected, such as hormonal deficiencies and bifid epiglottis as was seen in the patient in this case report. Faster identification of these issues may lead to improved outcomes. Making a clinical diagnosis of PHS is also important as these patients may be able to avoid some of the more aggressive measures required for other patients with hypothalamic hamartomas, such as AEDs and surgical resection. In pediatric patients with polydactyly and at least one of the sub-PHS criteria, MRI of the brain can easily identify a hypothalamic hamartoma and thus lead to an earlier and less invasive PHS diagnosis.

## References

[1] Hall JG, Pallister PD, Clarren SK (1980). Congenital hypothalamic hamartoblastoma, hypopituitarism, imperforate anus and postaxial polydactyly--a new syndrome? Part I: clinical, causal, and pathogenetic considerations. Am J Med Genet.

[2] Gorlin RJ, Cohen MMJ, Levin LS Syndromes of the Head and Neck. Oxford Univ Pr.

[3] Johnston JJ, Olivos-Glander I, Killoran C (2005). Molecular and clinical analyses of Greig cephalopolysyndactyly and Pallister-Hall syndromes: robust phenotype prediction from the type and position of GLI3 mutations. Am J Hum Genet.

[4] Biesecker LG (2000). Pallister-Hall Syndrome. Pallister-Hall Syndrome.

[5] Biesecker LG, Abbott M, Allen J (1996). Report from the workshop on Pallister-Hall syndrome and related phenotypes. Am J Med Genet.

[6] Kang S, Graham JM Jr, Olney AH, Biesecker LG (1997). GLI3 frameshift mutations cause autosomal dominant Pallister-Hall syndrome. Nat Genet.

[7] Chandra SR, Daryappa MM, Mukheem Mudabbir MA, Pooja M, Arivazhagan A (2017). Pallister-Hall syndrome. J Pediatr Neurosci.

[8] Watson BT, Hennrikus WL (1997). Postaxial Type-B polydactyly. Prevalence and treatment. J Bone Joint Surg Am.

[9] Mittal S, Mittal M, Montes JL, Farmer JP, Andermann F (2013). Hypothalamic hamartomas. Part 1. Clinical, neuroimaging, and neurophysiological characteristics. Neurosurg Focus.

[10] Carballo Cuello CM, De Jesus O (2021). Hypothalamic hamartoma. https://www.ncbi.nlm.nih.gov/books/NBK560663/.

[11] Boudreau EA, Liow K, Frattali CM (2005). Hypothalamic hamartomas and seizures: distinct natural history of isolated and Pallister-Hall syndrome cases. Epilepsia.

[12] Jung H, Neumaier Probst E, Hauffa BP, Partsch CJ, Dammann O (2003). Association of morphological characteristics with precocious puberty and/or gelastic seizures in hypothalamic hamartoma. J Clin Endocrinol Metab.

[13] Azzam A, Lerner DM, Peters KF (2005). Psychiatric and neuropsychological characterization of Pallister-Hall syndrome. Clin Genet.

[14] Arita K, Ikawa F, Kurisu K (1999). The relationship between magnetic resonance imaging findings and clinical manifestations of hypothalamic hamartoma. J Neurosurg.

[15] Rieth KG, Comite F, Dwyer AJ (1987). CT of cerebral abnormalities in precocious puberty. AJR Am J Roentgenol.

[16] Sanford RA, Muhlbauer MS (1991). Craniopharyngioma in children. Neurol Clin.

[17] Shah P, Patkar D, Patankar T, Shah J, Srinivasa P, Krishnan A (1999). MR imaging features in hypothalamic hamartoma: a report of three cases and review of literature. J Postgrad Med.

